# *In situ* observation of urothelial responses to NaCl-induced osmotic stress using optical coherence tomography

**DOI:** 10.1117/1.JBO.30.4.046009

**Published:** 2025-04-30

**Authors:** Lan Dao, Hui Zhu, Hui Wang

**Affiliations:** a Miami University, Department of Chemical and Biomedical Engineering, Oxford, Ohio, United States; b Cleveland Clinic Foundation, Glickman Urological and Kidney Institute, Department of Urology, Cleveland, Ohio, United States; c Louis Stokes Cleveland Department of Veterans Affairs Medical Center, Urology Section/Surgical Service, Cleveland, Ohio, United States

**Keywords:** optical coherence tomography, urothelium, permeability, osmolarity

## Abstract

**Significance:**

We provide the first direct evidence of the urothelial response to water transport through the urothelium, traditionally considered impermeable. Using optical coherence tomography (OCT), we observe that the urothelium absorbs and expels water under varying concentrations of NaCl, challenging long-held views about its impermeability. The discovery that osmotic stress can induce urothelial damage has implications for bladder disorders such as interstitial cystitis and overactive bladder, where urothelial integrity is compromised.

**Aim:**

Traditionally considered impermeable, the urothelium has recently been implicated in water transport due to the presence of aquaporins. Despite this, direct evidence of the urothelial response to water movement through the urothelium remains elusive. We aim to provide such evidence by examining urothelial responses to NaCl solutions using OCT.

**Approach:**

Fresh porcine bladder samples were subjected to OCT imaging to observe urothelial responses under varying osmolarity conditions, using NaCl solutions ranging from 0.31 to 2.07  Osm/L. Urothelial optical pathlength thickness was measured pre-NaCl and post-NaCl application. In addition, histological and scanning electron microscopy (SEM) analyses were conducted to assess cellular integrity and damage.

**Results:**

OCT imaging revealed a significant increase in urothelial optical pathlength thickness following deionized water application, indicative of water absorption. Conversely, exposure to higher osmolarity NaCl solutions resulted in urothelial shrinkage, suggesting water efflux. Histological analysis demonstrated intact cellular structures at lower osmolarities (0.31  Osm/L) but significant cellular disruption at higher concentrations (≥1.03  Osm/L). SEM analysis corroborated these findings, showing progressive damage to umbrella cells with increasing osmolarity.

**Conclusions:**

We provide evidence that the urothelium is a dynamic barrier capable of water transport, influenced by osmotic gradients. The observed osmotic-induced urothelial damage may have important implications for the pathophysiology of conditions such as interstitial cystitis and overactive bladder, offering insights into potential diagnostic and therapeutic strategies. These findings warrant further investigation using human tissue.

## 1 Introduction

The bladder is a round bag-like organ for storing urine and then expelling it under volitional control. The bladder wall consists of five layers of tissue, urothelium, lamina propria, submucosa, muscle, and fat. The urothelium is a thin layer lining the surface of the bladder wall formed by three cell layers, basal cell layer, intermediate cell layer, and umbrella cell layer. On the top of the umbrella cell layer, there is a thin glycosaminoglycan or GAG layer. The umbrella cells are closely packed together by tight junctions and covered with uroplakin, which provides strong protection for the urothelium.^1^ The diffusive permeability of water and ions measured with transepithelial electrical resistance and isotope tracer diffusion method seems very low.^2^^,^^3^ Therefore, conventionally, the urothelium has been considered a physical barrier between the urine and the “blood” to protect the underlying bladder tissues, against urine, toxins, and infections such as bacteria.

The concept of the impermeability of the urothelium has been challenged from the very beginning of its development. Almost a century ago, an *in vivo* animal study demonstrated water and sodium chloride (NaCl) transport through the urothelium by tracking the concentration variations of the added hemoglobin and chloride ions.^4^ In a later *in vivo* human study, water absorption was reported by tracking tritiated water.^5^ With the advancements in molecular biology, researchers have discovered the expression of aquaporins (AQPs), a family of water channel proteins, on the urothelium of a variety of species, including humans. AQP-3, -4, -7, and -9 have been identified on human surgical tissue and differentiated normal human urothelial cells^6^; AQP-1, -2, and -3 have been identified on rat bladder, and AQP-1, -3, -9, and -11 have also identified on the porcine bladder.^7^^,^^8^ These discoveries provided the molecular mechanism for water regulation and transport through the urothelium. With ultrasonic imaging, the bladder volume of a group of participants was monitored while sleeping.^9^^,^^10^ Significant volume reduction of the urinary volume indicated water transportation in the bladder. The observation was further substantiated by monitoring the change in the concentration of a blue dye, which correlated with the alternation of bladder volume. In cultured differentiated human urothelium, NaCl solutions were found to significantly upregulate AQP3 expression by over 10-fold, and preconditioning with hyperosmotic NaCl resulted in a 3.6-fold increase in water flux across the urothelium under an osmotic gradient, as measured using radioactive tracers and permeability coefficients.^11^ Similar results were observed in rat and porcine bladders.^12^^,^^13^

Over the past century, various technologies have been used to investigate the permeability of the urothelium. The microstructures observed on umbrella cells suggest that the urothelium serves as a protective barrier for the underlying tissue, whereas the presence of aquaporins (AQPs) throughout the urothelium indicates a potential role in regulating water and ion transport. Although tracers can track the movement of water molecules, the mechanisms by which the urothelium responds to water transport remain unclear. Optical coherence tomography (OCT) is a noninvasive imaging technology, which can acquire tissue cross-sectional images at a resolution of less than 10  μm. OCT has been developed for urological cancer diagnosis as it can clearly differentiate the urothelium, the lamina propria, and the detrusor muscle.^14^^,^^15^ In this report, using OCT, for the first time, we demonstrate *in situ* observation of the urothelial responses to NaCl solution under different osmatic concentrations due to water transport.

## 2 Materials and Methods

### 2.1 OCT Imaging

An in-house-built spectral-domain OCT system was used for acquiring OCT images. A supercontinuum light source (YSL Photonics, Mill Valley, California, United States) filtered at ∼850  nm with ∼150  nm full width at half maximum was used as the broadband light source. The axial resolution is ∼2  μm, and the lateral resolution is ∼7.9  μm, with a power of ∼2.3  mW on the sample. OCT images were acquired at 17 frames per second with 1000 A-scans per frame. The raw OCT data were postprocessed in MATLAB and saved as 500×500-pixel images for further processing. The details of the hardware and the software were described in our previous publication.^16^

### 2.2 Tissue Preparation

**Fig. 1:**
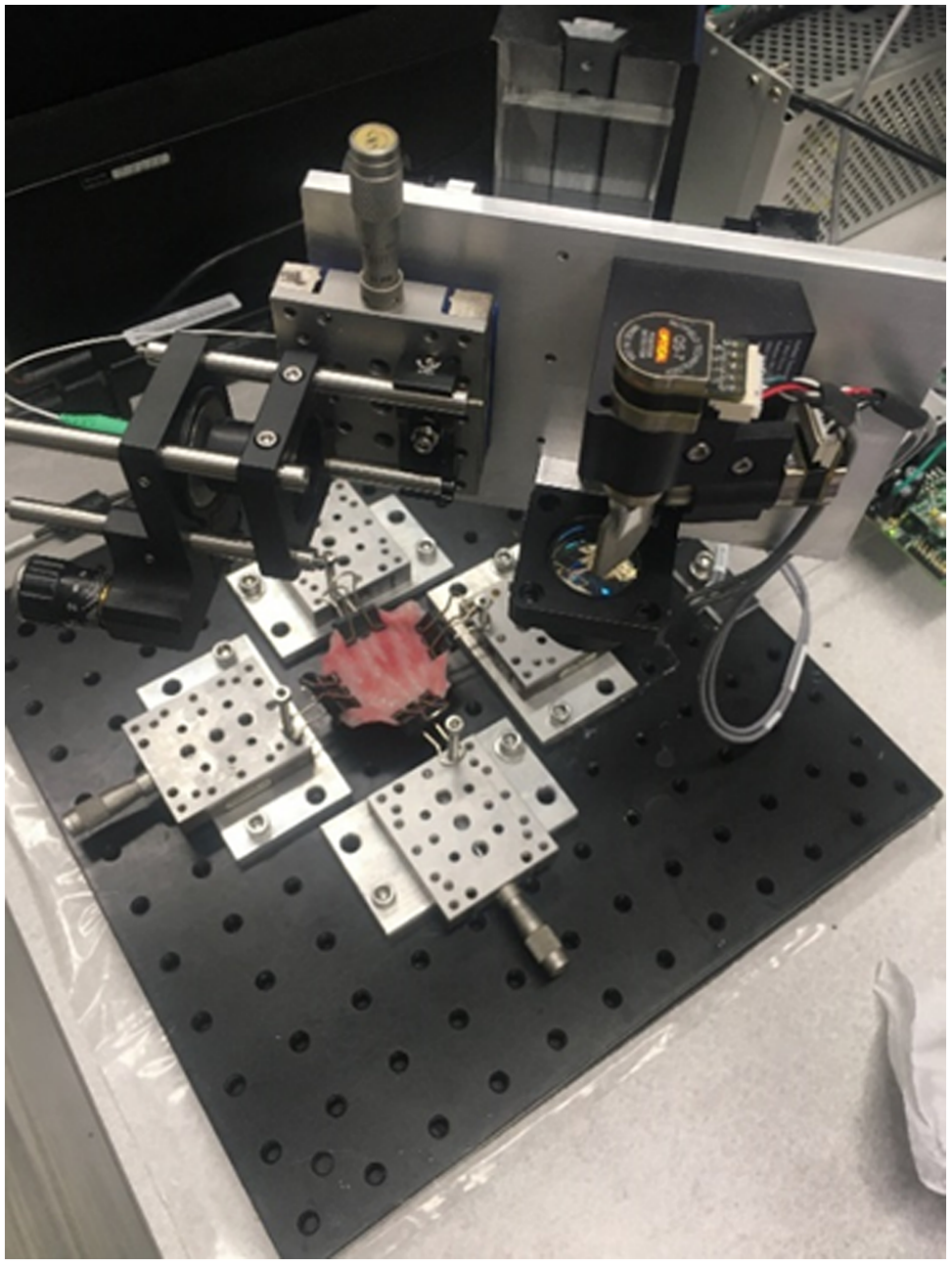
Tissue bench used for mounting bladder tissue samples, enabling uniform stretching of the bladder using four precisely controlled translation stages.

Fresh porcine bladders were collected from a local slaughterhouse immediately after the animal sacrifice. The collected porcine bladders were transported and stored in Krebs–Henseleit solution at 4°C. All experiments in this study were completed in 6 h.^17^^,^^18^ The bladder was first gently washed with deionized (DI) water and then cut into four 3  cm×3  cm specimens. The four edges of a tissue sample were mounted by clippers on four translational stages. During imaging, the tissue sample was gently and uniformly stretched by the translational stages from all edges so that the urothelium layer could be clearly visualized under OCT. The setup of the tissue bench is shown in Fig. 1.

### 2.3 Urothelial Thickness Measurement

A precision microscope cover slide (CG15CH2, 170±5  μm, Thorlabs, Newton, New Jersey, United States) was used to calibrate the thickness measurements of the urothelium from OCT images. The refractive index of the glass slide at 850 nm was determined to be 1.51 using the Zemax material database. An OCT image of the cover slide was acquired with the identifiable bottom and top surfaces of the slide. The calibration factor, optical path thickness (OPT) per pixel, is calculated as

$$\text{Calibration factor }(\frac{\mathrm{OPT}}{\text{pixel}})=\frac{170\;\mu m\;(\text{the thickness of the slide})\times 1.51\;(\text{refractive index})}{\text{The number of pixels between bottom and top surfaces}}.$$ (1)

The upper and lower urothelial boundaries were first identified in OCT images. The pixel count between these boundaries was measured with ImageJ. Next, we multiplied the pixel count by the calibration factor (2.7±0.15  μm/pixel) to determine the OPT of the urothelium. Physical thickness can be estimated by taking the ratio of the OPT to the urothelium’s refractive index. However, because the refractive index of biological tissue can fluctuate with hydration status,^19^ we report urothelial thickness in terms of OPT.

### 2.4 Urothelial Thickness Variation

After a tissue sample was mounted, the tissue sample was first gently dried by blowing off the residual water on the surface with air for 10 s. Then, DI water was added. OCT recorded the urothelial response while adding the DI water. For observing the urothelial response at different osmolarity concentrations, the tissue surface was first covered with a thin layer of DI water, and then, 100  μL 2.07  Osm/L (6%) NaCl was added to the imaged site to replace the DI water.

### 2.5 Urothelial Thickness Quantification at Different Osmolarities

**Fig. 2:**
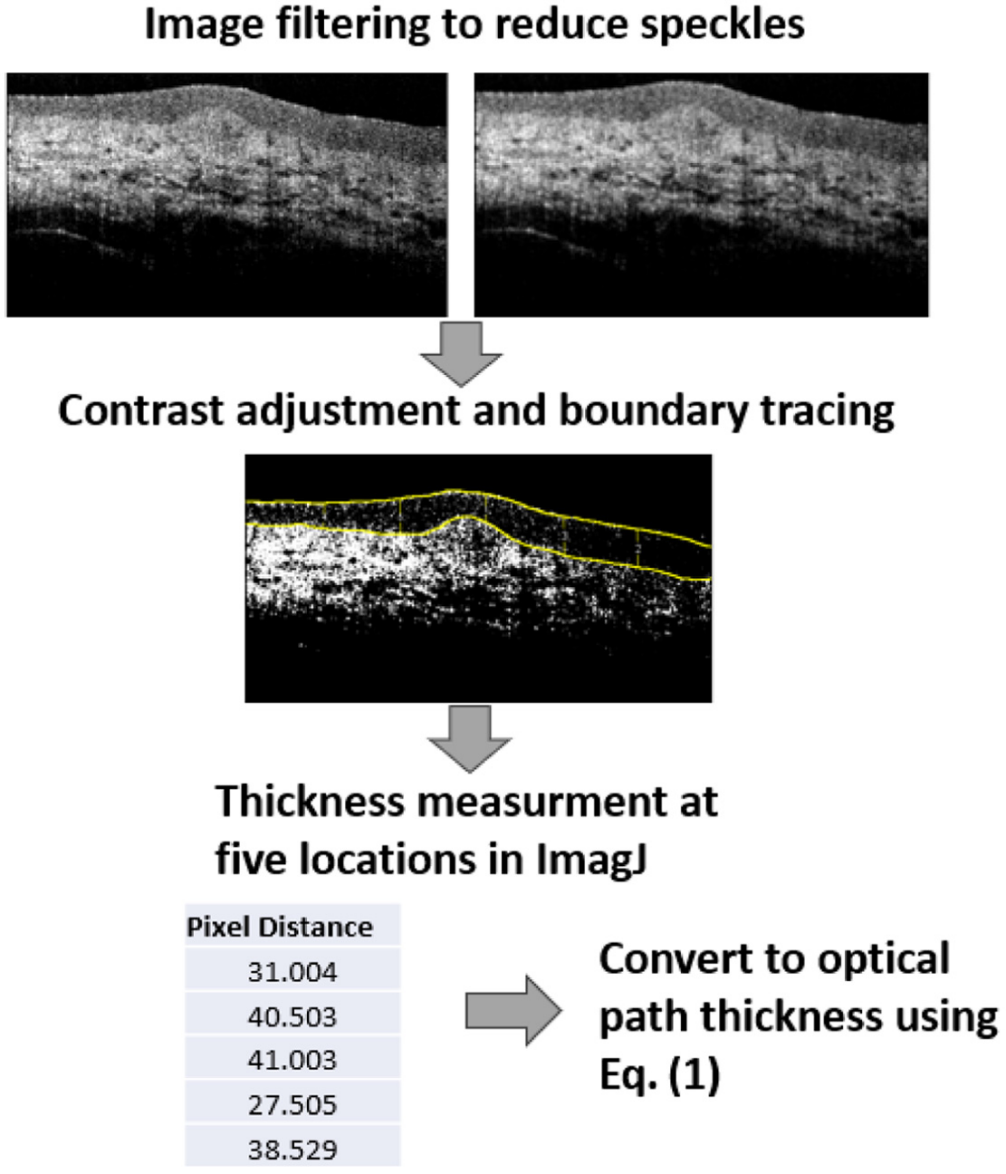
Image processing and OPT quantification flow.

After a tissue sample was mounted, 100  μL of 0.31  Osm/L (0.9%), 0.52  Osm/L (1.5%), 0.69  Osm/L (2%), 0.86  Osm/L (2.5%), 1.03  Osm/L (3%), 1.38  Osm/L (4%), and 2.07  Osm/L (6%) NaCl solution was gently and sequentially added to the surface of a tissue sample. The tissue surface was washed with DI water, and the surface residual water was dried with a Kimwipe before adding the solution with different concentrations. The OCT recorded tissue responses through the solution-adding process. Seven tissue samples, each from a different bladder, were analyzed for each osmolarity. Figure 2 illustrates the quantification process flow for measuring urothelium OPT. All processing and measurements were conducted using ImageJ. First, OCT images were smoothed to reduce speckle patterns using the smooth function (3×3 mean filter) of ImageJ. The contrast was then adjusted to create a black-and-white image, allowing for clear boundary tracing between the urothelium and the lamina propria. The top surface and boundary were manually traced. OPT measurements were taken at five horizontal pixel positions: 83, 166, 250, 333, and 416. The measured OPT, recorded in pixels, was then converted to OPT using Eq. (1). Additional information is available in the Supplementary Material.

### 2.6 Urothelial Damage at Different Osmolarity

Each bladder was initially divided into six tissue samples. Each sample was mounted on a tissue bench and stretched to twice its original dimensions in two orthogonal directions, as performed during OCT imaging. The samples were then treated by covering the tissue surface with one of the six NaCl solutions, with osmolarities of 0  Osm/L (water), 0.31  Osm/L (0.9%), 0.69  Osm/L (2%), 1.03  Osm/L (3%), 1.38  Osm/L (4%), and 2.07  Osm/L (6%), for 10 min. Following treatment, the tissue samples were dried using the previously described method and cut into three blocks. Two blocks were fixed in 10% formalin for hematoxylin and eosin (H&E) staining, whereas the third block was fixed for scanning electron microscopy (SEM) imaging. For H&E analysis, two histological slices were prepared from each tissue block, yielding a total of 40 histological slides for each NaCl concentration across 10 different bladders. A total of 240 slides were analyzed.

## 3 Results

**Fig. 3:**
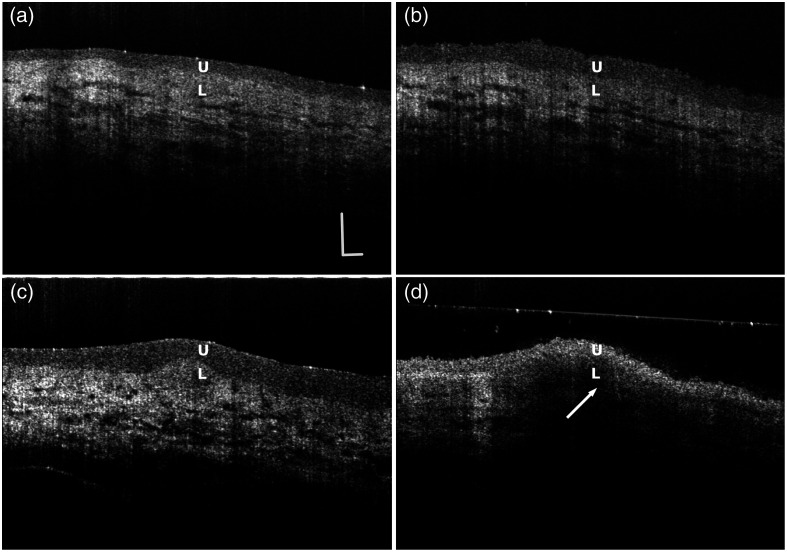
*In situ* monitoring urothelial OPT change under OCT. (a) and (b) Urothelial OPT change before and after adding DI water. (c) and (d) Urothelial OPT change before and after adding 0.207  Osm/L NaCl solution. U, urothelium; L, lamina propria (scale bar: 100  μm) (Video 1, AVI, 3.74 MB [URL: https://doi.org/10.1117/1.JBO.30.4.046009.s1]; Video 2, AVI, 2.37 MB [URL: https://doi.org/10.1117/1.JBO.30.4.046009.s2]).

In the OCT images, the urothelium appears as a layer with relatively lower contrast atop the lamina propria, which exhibits stronger contrast. As OCT contrast originates from backscattered light, it is directly related to the tissue’s scattering properties. Figures 3(a) and 3(b) illustrate the changes in urothelial OPT before and after the application of DI water to a dried urothelial surface. The OPT of the urothelium increased by ∼52% following the addition of DI water, indicating significant water absorption by the urothelial cells. Figures 3(c) and 3(d) show the urothelial OPT before and after applying a 2.07  Osm/L NaCl solution to a tissue sample. A notable shrinkage of the urothelium is observed between Figs. 3(c) and 3(d), suggesting that intracellular water was expelled from the urothelial layer due to the osmolarity gradient. As a result of the water extraction, the cellular organelles within the urothelium were densely packed, enhancing light scattering observed under OCT. The arrow in Fig. 3(d) points to a shadow beneath the urothelial layer, indicating that the intense scattering from the contracted urothelium obstructs the light backscattered from the lamina propria. As a result, the contrast between the urothelium and the lamina propria becomes diminished in some regions. The processes of the urothelial response can be watched in Videos 1 and 2.

**Fig. 4:**
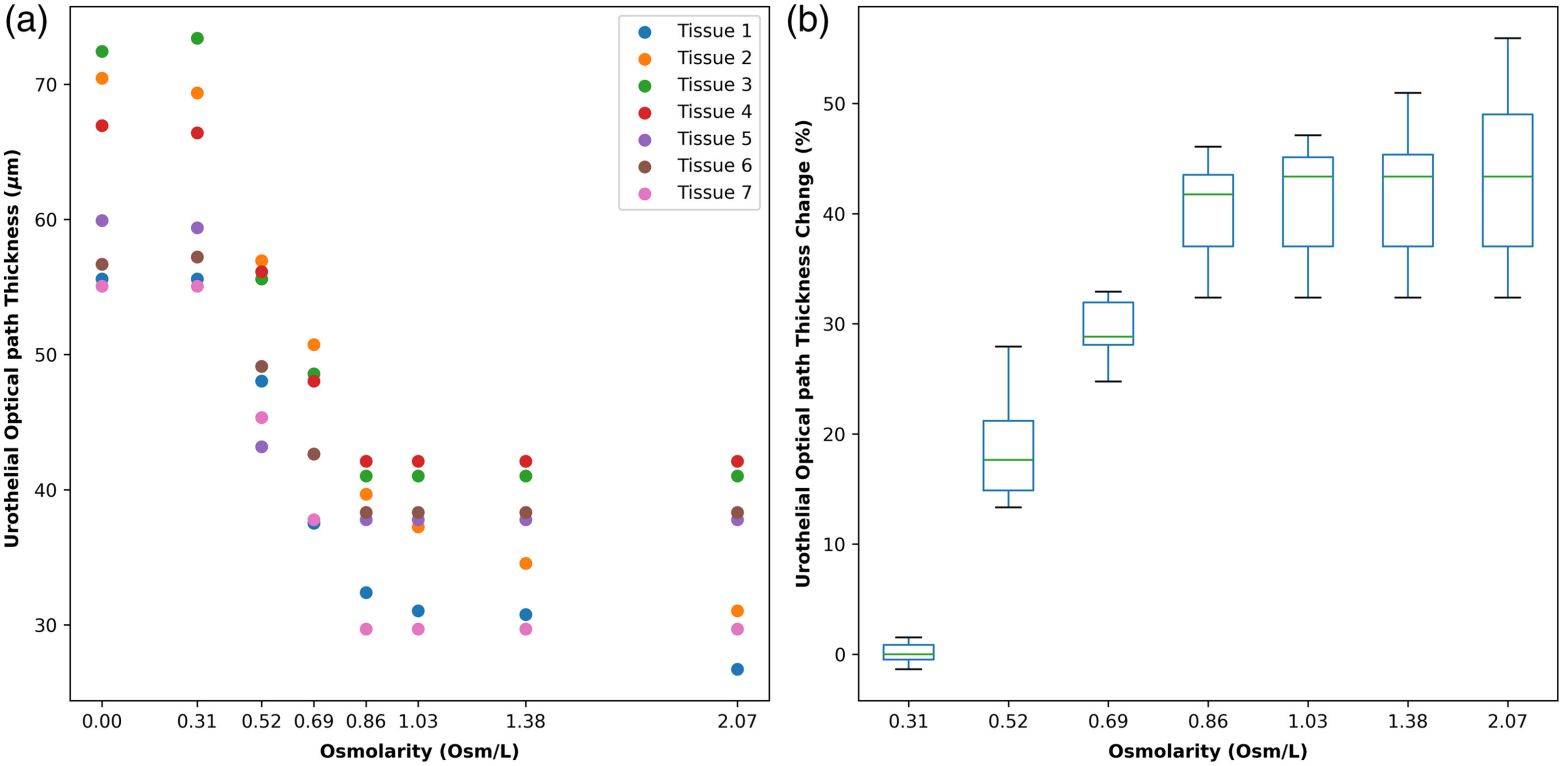
Urothelial OPT variation after applying NaCl solutions at different osmolarities. (a) The change of the urothelial OPT of seven tissue samples. (b) The percentage change of the urothelial OPT of seven tissue samples.

To quantify the urothelial responses treated with NaCl solution at different osmolarities, the urothelial OPT variation was measured using OCT images. As plotted in Fig. 4(a), the urothelial OPT change is plotted against eight discrete osmolarities. The experiment was repeated on seven tissue samples from different bladders, yielding an average standard deviation of 11%. Figure 4(b) further plots the percentage change in urothelial OPT in comparison to its initial OPT when treated with DI water. When the osmolarity is equivalent to normal saline (0.31  Osm/L), urothelial OPT exhibits negligible changes from the baseline. However, as the osmolarity is increased to 0.52  Osm/L, the OPT is decreased by ∼20%. This contraction can reach around 30% at an osmolarity of 0.69  Osm/L and escalate to 40% with a further increased osmolarity to 0.86  Osm/L. Beyond this concentration, further increasing NaCl osmolarity does not appear to induce additional urothelial shrinkage. The observed decrement in urothelial OPT is indicative of water efflux from the urothelium, presumably triggered by the osmotic gradient established between intracellular and extracellular environments.

**Fig. 5:**
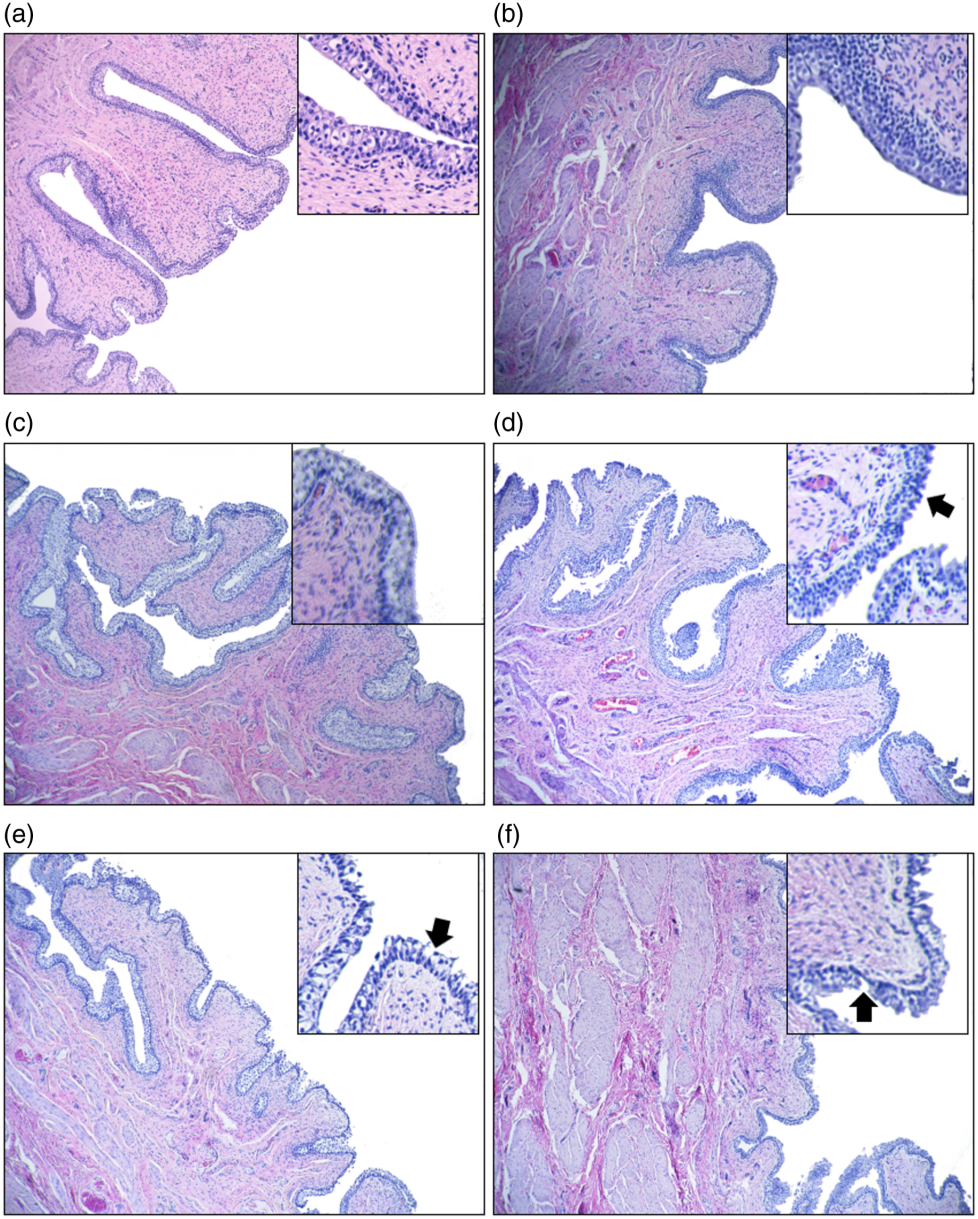
H&E (10×) of the tissue samples after applying NaCl with different osmolarities, including zoomed-in insets for detailed visualization. (a) DI water; (b) 0.31  Osm/L (saline); (c) 0.69  Osm/L; (d) 1.03  Osm/L; (e) 1.38  Osm/L; and (f) 2.07  Osm/L. The black arrows indicate the damage to the urothelium.

Histological analysis was performed using H&E staining to assess cellular-level responses in tissue samples exposed to NaCl solutions of varying osmolarities. Figure 5 shows representative H&E images from 240 slides. The urothelium in samples treated with DI water and saline (0.31  Osm/L) remained largely intact, displaying distinct layers, including the umbrella cell layer, intermediate cell layer, and basal cell layer, in Figs. 5(a) and 5(b). Although the sample exposed to 0.69  Osm/L NaCl exhibited an ∼30% reduction in urothelial OPT as shown in Fig. 4(b), no overt damage was observable in the H&E image as shown in Fig. 5(c). More pronounced damage was noted in the sample treated with 1.03  Osm/L NaCl, particularly in the umbrella cell layer, where discontinuities appeared on the surface of the urothelium indicated by the black arrow in Fig. 5(d). Increasing the NaCl concentration to 1.38 and 2.07  Osm/L resulted in severe damage throughout the urothelial layers; at some locations, both the umbrella and intermediate cell layers were completely lost indicated by the black arrow in Figs. 5(e) and 5(f).

**Fig. 6:**
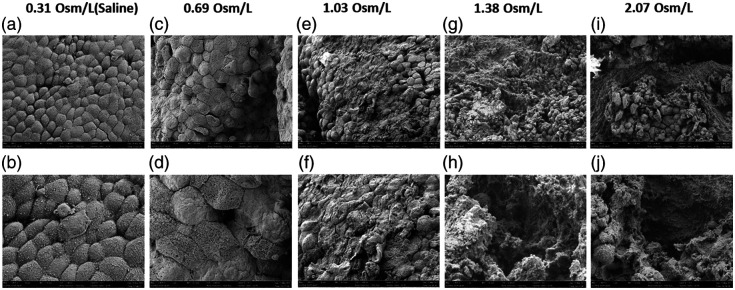
Scanning electron microscope images of the tissue samples treated with NaCl at different osmolarities. (a) and (b) Saline; (c) and (d) 0.069  Osm/L; (e) and (f) 0.103  Osm/L; (g) and (h) 0.138  Osm/L; (i) and (j) 0.207  Osm/L.

SEM has been used to image the ultrastructure of the urothelial cells of the tissue samples treated with NaCl solutions. For the tissue samples treated with saline, intact dome-shaped umbrella cells covered with asymmetric unit membrane particles can be identified [Figs. 6(a) and 6(b)]. This observation is aligned with the H&E image [Fig. 5(b)], where the umbrella cell layer is complete. For the tissue sample treated with NaCl solution at 0.69  Osm/L, the urothelium looks intact from the H&E image [Fig. 5(c)]. However, on the SEM images [Figs. 6(c) and 6(d)], it seems that the umbrella cells are still complete, but the surface of the umbrella cells has been subtly damaged. For the tissue sample treated with NaCl at 1.03  Osm/L, the damage on the cell membrane of the umbrella cells is visible [Figs. 6(e) and 6(f)]. Accordingly, such damage also appears in the histology image [Fig. 5(d)]. With the increased osmolarity, from SEM images shown in Figs. 6(h)–6(j), severe damage to the urothelial layer can be observed. The loss of the entire umbrella cell layer exposes the below basal cell layers or even the lamina propria. A similar level of damage has also been observed in the corresponding histological images [Figs. 5(e) and 5(f)]. The SEM images confirm histology analysis observation and reveal some damage to the ultrastructure on the umbrella cells, which cannot be identified through histology.

## 4 Discussion and Conclusion

Historically, the urothelium has been examined through two primary perspectives: the impermeable barrier theory and the permeable barrier theory. The impermeable barrier theory posits that the urothelium functions as a robust physical barrier, preventing the passage of water, ions, and pathogens. This theory is supported by the structural integrity of umbrella cells, tight junctions, and the uroplakin layer, which together form a formidable defense against urinary components and pathogens. The permeable barrier theory, which has gained traction with advancements in molecular biology, suggests that the urothelium is selectively permeable and capable of regulating water and ion transport through specialized channels and proteins. Key among these are AQPs, a family of water channel proteins that facilitate the rapid transport of water across cell membranes. AQPs, such as AQP-3, -4, -7, and -9 in humans, have been identified in the urothelium, indicating a molecular basis for water permeability.^6^ In addition, *in vivo* observations of bladder volumetric changes during sleep provide further evidence that the bladder can regulate water.^9^^,^^10^

If water can be transported through the urothelium, the most relevant evidence would be observing how the urothelium responds to water transport. OCT, a nontissue invasive imaging modality, enables us to visualize this process. Although the imaging depth of OCT is limited to 1 to 2 mm, its resolution can reach a few micrometers. In urology, OCT has been developed for the early diagnosis of bladder and urethral cancers.^20^^,^^21^ When appropriately stretched, the bladder shows a layered structure where the urothelium, lamina propria, and muscularis layer can be visualized. Thus, OCT can be used to observe the urothelial response to water transport.

The *in situ* observation of the urothelial response to NaCl solution provides new insights into the permeability and structural integrity of the urothelium under varying osmotic conditions. Water transport is typically bidirectional. In Fig. 3(a) and Video 1, the urothelial OPT increased by ∼52% after the application of DI water over surface-dried tissue, indicating substantial water absorption by urothelial cells. The water absorption aligns with the observed reduction in bladder volume during sleep.^9^^,^^10^ Conversely, exposure to high-osmolarity NaCl solution (2.07  Osm/L) resulted in shrinkage of the urothelium. This osmotic challenge caused water efflux from the urothelial cells, leading to cellular dehydration and increased packing density of cellular organelles. The enhanced light scattering observed in OCT images, indicated by the shadow beneath the urothelial layer, underscores the structural changes occurring due to osmotic stress, as shown in Fig. 3(b) and Video 2. Osmolarity appears to play a critical role in water transport.^22^ In the second study, we conducted a quantitative analysis of urothelial OPT at varying osmolarities. Although normal saline (0.31  Osm/L) caused negligible changes, higher osmolarities led to significant contraction of the urothelium. The OPT reduction plateaued beyond 0.86  Osm/L, suggesting that most of the free water had been expelled from the urothelial cells due to osmotic pressure.

Histological analysis allows us to observe changes in tissue microstructures due to significant water transport. The urothelial layer remains intact when treated with DI water and normal saline. However, increased osmolarity results in progressive damage, particularly in the umbrella cell layer. The damage is most pronounced at higher osmolarities, where both the umbrella and intermediate cell layers are severely affected. SEM images further confirm these observations, revealing ultrastructural damage not visible in histological images. The subtle damage to the umbrella cells at lower osmolarities and severe damage at higher osmolarities highlight the sensitivity of the urothelium to osmotic changes. The loss of the umbrella cell layer and exposure of underlying tissues at high osmolarities indicate significant disruption of the urothelial barrier. The damage to the urothelial cells could be related to osmotic lysis; when water exits the cells to balance the osmotic pressure in a short period, the sudden cell shrinkage may lead to cell rupture.^23^

The findings from this study may have implications for conditions such as interstitial cystitis/painful bladder syndrome (IC/PBS) and overactive bladder (OAB). IC/PBS and OAB are complex disorders. IC/PBS is characterized by chronic pelvic pain, pressure, or discomfort associated with lower urinary tract symptoms, in the absence of infection or other identifiable causes, whereas OAB was defined as a syndrome as “urinary urgency, with or without urgency urinary incontinence, usually with increased daytime frequency and nocturia, if there is no proven infection or other obvious pathology.”^24^^,^^25^ The etiology of IC/PBS and OAB remains elusive, with various hypotheses proposed but none conclusively proven in clinical studies. For IC/PBS, a predominant theory suggests that urothelial damage induces bladder pain. This hypothesis assumes that compromised urothelial integrity increases permeability to concentrated urinary electrolytes, leading to inflammation and pain via activation of afferent sensory neurons in the bladder’s lamina propria. For OAB, there is a theory/hypothesis that describes the urgency that originated from the urothelium/suburothelium due to abnormal sensory function within the urothelium/suburothelium. However, it is not clear yet what causes urothelial damage.

In our study, with porcine bladder tissue, we observed damage to umbrella cells within minutes of exposure to NaCl solution. Previous studies have linked the permeability of urothelial cells to osmatic stress, especially NaCl. In a porcine bladder model, water flux across the urothelium was significantly reduced in the presence of the AQP inhibitor mercuric chloride, demonstrating that AQPs mediate transcellular water transport under osmotic stress.^13^ It has also been found that in differentiated human urothelial cells, AQP-3 is significantly upregulated by increased NaCl osmolality, but not by urea and glucose.^11^ In rats, AQP-2 expression on urothelial cells is significantly higher after injecting saline into the bladder compared with glucose. High salt intake has been shown to induce OAB-like symptoms in animal models.^26^^–^^28^ Interestingly, clinical studies have demonstrated improved urinary symptoms in OAB patients following salt intake restriction.^29^ These observations suggest that NaCl may play an important role in regulating water transport through urothelial cells, although the mechanisms have not been disclosed.

Under SEM, umbrella cell damage is observed at an osmolality of 0.69  Osm/L, a concentration that exceeds typical sodium levels in human urine.^30^ Our key finding is osmotic lysis resulting from substantial water transport, which has been shown in previous studies to be driven by upregulated AQPs and osmotic stress. In this context, NaCl may play a dual role: upregulating AQPs and creating osmotic stress, leading to significant water transport. Beyond NaCl, AQP expression can be influenced by various physiological factors. In rats, dehydration upregulates AQP-2 and AQP-3, bladder distension from mechanical stress increases AQP-2, and pathological conditions such as detrusor overactivity caused by partial bladder outlet obstruction upregulate AQP-1.^8^^,^^31^ In addition, factors such as vitamin D, intermittent fasting, and high-fat diets can modulate AQP-1 and expression.^32^ Hormonal influences, such as vasopressin, a known regulator of AQP expression in renal tissues, may exert similar effects in the bladder.^33^ With increased AQPs and high osmotic stress due to urine osmolality, which can reach up to ∼1.2  Osm/L,^34^ water efflux into urothelial cells can lead to cell damage or lysis. The diverse mechanisms of AQP upregulation may also suggest the complex phenotypes observed in IC/PBS and OAB. However, this hypothesis requires rigorous validation through further research. It will be interesting to use endoscopic OCT to monitor the urothelial response to osmatic stress in patients with hormonal-related diseases, such as diabetes mellitus, in the future.

Creating controlled urothelial damage in animal models is also important for researchers to investigate physiological processes such as inflammation, cell death, regeneration, and scar formation in the bladder. Previously, such damages were induced by injecting toxic chemical agents (e.g., protamine sulfate).^35^^,^^36^ Here, we show an easy and safe method to create progressive urothelial damage using NaCl solution at different osmolarities.

The present study has several limitations that warrant further investigation. First, all experiments were conducted *ex vivo* using porcine bladder tissue. Although this model provides valuable insights, it does not fully replicate the complex physiological conditions present *in vivo*, such as blood flow and hormonal regulation, that are absent in *ex vivo* studies. Future research should begin with *in vivo* experiments in large animal models. The porcine bladder, due to its anatomical and physiological similarities to the human bladder, has proven to be an excellent model for studying bladder cancer.^37^ These similarities enable the use of clinical tools and techniques directly transferable to human applications. Integrating OCT with cystoscopy offers a promising approach to observe urothelial responses to osmotic stress in live large animal models.^38^^,^^39^ However, findings from porcine tissue studies must be validated using human bladder tissue, such as differentiated human urothelial cells, to bridge the gap between preclinical research and clinical applications. These verifications are crucial before advancing to clinical trials. By following this pathway, the technology could be adapted by urologists to investigate the underlying mechanisms of IC/BPS and OAB, ultimately enhancing diagnostic and therapeutic strategies for these conditions.

## Supplementary Material

10.1117/1.JBO.30.4.046009.s01

10.1117/1.JBO.30.4.046009.s1

10.1117/1.JBO.30.4.046009.s2

## Data Availability

The raw data study will be provided upon request to the corresponding author.
