# Supplementary material for: In situ observation of urothelial responses to NaCl-induced osmotic stress using optical coherence tomography
Source: J Biomed Opt. 2025 Apr 30;30(4):046009. doi: 10.1117/1.JBO.30.4.046009 (PMC12041670; doi:10.1117/1.JBO.30.4.046009)
Supplement: Supplementary file 1 [file JBO_030_046009_SD001.docx]

**Supplemental Material**

**Detail description of measuring OPT of the urothelium**

1. **Image Smoothing:** The OCT images were smoothed in ImageJ to reduce the impact of speckle noise.

Left: the original image Right: the smoothed images

| 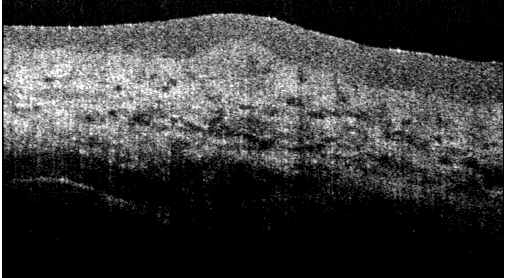 | 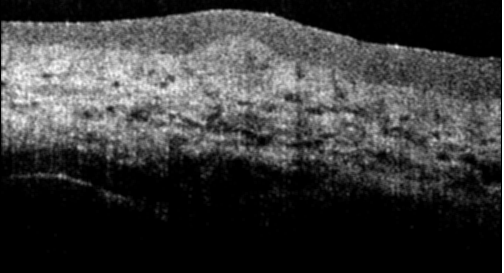 |
| --- | --- |
| 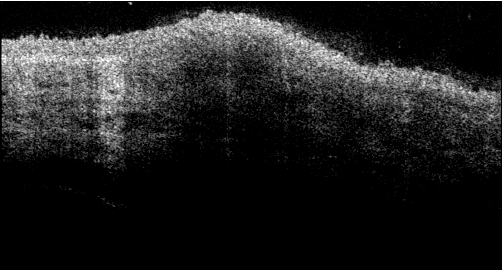 | 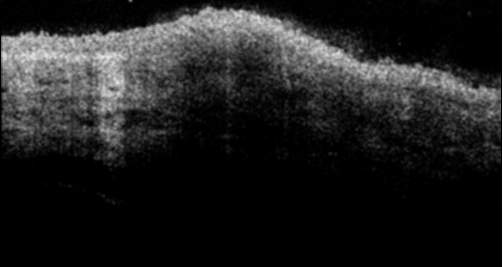 |

1. **Contrast Adjustment and Boundary Tracing:** We adjusted the contrast to clearly delineate the boundary between the urothelium and the lamina propria—a step analogous to the threshold selection process used in automated segmentation. Notably, the contrast of the urothelium is inverted to white as illustrated in the right-hand figure below, due to the significant compression caused by the application of a highly osmotic NaCl solution. Additionally, in the region with strong scattering (indicated by the arrow), we intentionally avoided this area by assuming a smooth boundary.

| 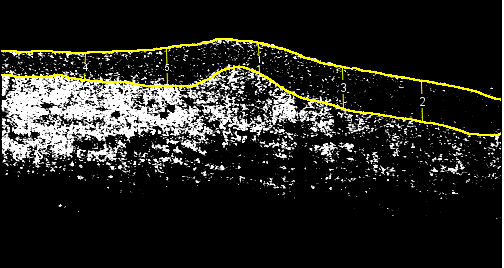 | 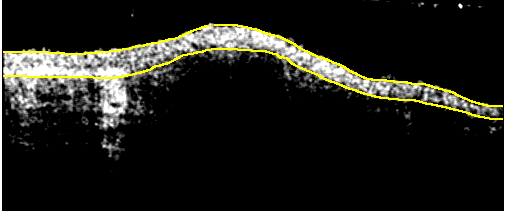 |
| --- | --- |

1. **Thickness Measurement:** Five lines were drawn between the boundaries at pixel positions 83, 166, 250, 333, and 416 along the horizontal axis, and the pixel distances were measured using ImageJ. The measured distances were directly obtained from the "Length" column in ImageJ, as shown in the figure below.

| 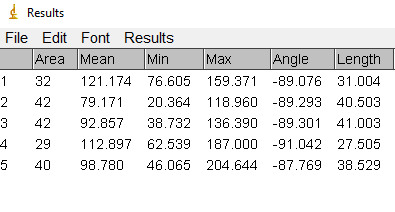 | 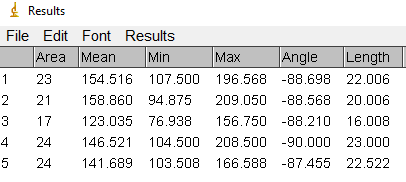 |
| --- | --- |

1. **Conversion to Optical Path Length:** The measured pixel distances were converted to OPT as:

OPT = Calibration Factor × Pixel Distance
